# Optimizing Transcutaneous Electrical Stimulation Based on Frequency-Dependent Tissue Modeling and Signal Analysis

**DOI:** 10.3390/bioengineering13080847

**Published:** 2026-07-23

**Authors:** Wenzhu Wu, Junquan Tang, Qiong Wang, Jun Yang

**Affiliations:** 1 Key Laboratory of Brain-Machine Fusion and Intelligent Medical Equipment, Basic Courses Department, Chongqing Medical and Pharmaceutical College, Chongqing 401331, China; 10742@cqmpc.edu.cn (W.W.); 10769@cqmpc.edu.cn (J.T.); 2 Key Laboratory of Biorheological Science and Technology, Ministry of Education, Bioengineering College, Chongqing University, Chongqing 400044, China; bioyangjun@cqu.edu.cn

**Keywords:** transcutaneous electrical stimulation, finite element modeling, frequency-dependent tissue properties, waveform optimization, electrode configuration, penetration ratio

## Abstract

Transcutaneous electrical stimulation (TES) is limited by cutaneous discomfort caused by unavoidable activation of superficial sensory nerves during current delivery to deep targets. While psychophysical studies have empirically identified waveforms that reduce skin sensation, existing computational models typically employ quasi-static approximations that neglect the pronounced dielectric dispersion of biological tissues, leaving the biophysical mechanisms poorly understood. We developed a finite-element model of the human forearm incorporating the frequency-dependent dielectric properties of skin, fat, muscle, bone, and nerve tissues (DC to 1 MHz), coupled with a linear time-invariant signal-processing framework based on the system transfer function *H*(*f*). The model quantitatively reproduced the waveform-dependent sensation trends reported by Hsu et al. We introduced a penetration ratio to quantify deep-to-superficial nerve activation and found that all time-varying waveforms exhibit lower penetration than direct current (DC), revealing a skin-effect-like behavior of electrical current in biological tissues. Moreover, deep and superficial nerve activations co-varied under waveform parameter changes, indicating that waveform optimization alone cannot improve depth selectivity. Electrode spatial configuration was shown to offer a complementary strategy: positioning the active electrode close to the target muscle nerve while avoiding superficial cutaneous nerves, combined with sufficient transverse spacing, enhances depth selectivity. This work bridges psychophysical observations with tissue electrophysiology and provides a computational tool for waveform and electrode optimization in TES.

## 1. Introduction

Transcutaneous electrical stimulation (TES) is a non-invasive neuromodulation technique that delivers controlled current through surface electrodes to modulate the activity of peripheral nerves, spinal circuits, or cortical regions [1,2]. Depending on the stimulation target and purpose, TES encompasses several modalities, including transcutaneous electrical nerve stimulation (TENS) for pain management [3], functional electrical stimulation (FES) for motor rehabilitation [4], and transcutaneous spinal cord stimulation (tSCS) for spinal circuit modulation [5]. These modalities have been widely applied in chronic pain management, stroke rehabilitation, and spinal cord injury rehabilitation. TES faces a fundamental challenge, however: the stimulating current must pass through the skin before reaching deep target nerves, and in doing so, it inevitably activates superficial sensory nerves, causing discomfort such as prickling, burning, and itching that directly limits the tolerable stimulation intensity and reduces therapeutic efficacy [6]. How to effectively activate deep target nerves while reducing superficial sensation remains a core problem of persistent concern in the TES field.

To understand the relationship between stimulation waveforms and cutaneous sensation, Hsu et al. systematically compared the perceived intensity of monophasic square waves, monophasic sine waves, and amplitude-modulated (AM) sine waves on the human forearm [7]. They found that constant DC produced the weakest sensation; increasing duty cycle or frequency reduced sensation but never below the DC level; for biphasic AM waves, higher carrier frequencies and lower amplitudes yielded weaker sensations. These results provide an important empirical basis for waveform selection, but the underlying biophysical mechanisms remain unclear. Proposed hypotheses include low-pass filtering properties of sensory axon membranes [8] and frequency-dependent tissue dielectric properties [9,10]; yet, a computational model capable of quantitatively predicting neural activation strength for different waveforms has been lacking.

Computational modeling has become an important tool for optimizing TES. In electrode configuration optimization, Javaid et al. [4] and Ota et al. [11] used finite element simulation to optimize TENS electrode spacing and placement, respectively, while Xiao et al. [12] and Parazzini et al. [13] optimized electrode structures and arrangements for transcranial stimulation. Regarding neural activation metrics, the activating function, the second spatial derivative of the extracellular potential along the nerve axis, has been shown to be a more direct predictor of neural depolarization than electric field magnitude [14,15]. Cassar et al. [16] used it to optimize concentric electrode design for FES, and Iseri et al. [17] employed a similar approach to determine current density thresholds for transcorneal electrical stimulation. In novel stimulation modalities, temporal interference (TI) stimulation uses two kilohertz-range currents to generate a low-frequency envelope at depth; Xie et al. [18] and Teragiwa et al. [19] verified its depth selectivity advantage in spinal cord stimulation through simulation. Gomez–Tames and Fernandez–Corazza reviewed 57 transcranial electrical stimulation computational studies and noted that the field is moving toward individualized electric field analysis and lead optimization [20].

Most of these computational studies, however, employ quasi-static approximations or single-frequency tissue conductivity values [3,5,12], without accounting for the significant dielectric dispersion of biological tissues over the DC-to-1 MHz range. Within this frequency band, conductivity and permittivity can vary by several orders of magnitude [9,10], fundamentally altering the current distribution of different frequency components. When comparing stimulation waveforms that contain different frequency components, ignoring this dispersion may lead to prediction bias. In terms of neural activation metrics, the activating function has been widely used to predict neural depolarization: Rattay [14] first demonstrated that the excitation effect of external electric fields on axons is primarily determined by this second derivative; McIntyre and Grill [15] extended it to non-uniform electric field conditions, and recent high-frequency interference stimulation research [21] has reaffirmed its applicability under complex field configurations. To this end, we constructed a finite element model of the human forearm containing skin (stratum corneum and dermis), fat, muscle, bone, blood vessels, and major nerves, with frequency-dependent dielectric properties assigned to all tissues (DC to 1 MHz). In the frequency-domain analysis, the activating function for each nerve was computed under unit current excitation; the system transfer function *H*(*f*) was then defined as the ratio of this activating function to the input current. *H*(*f*) fully characterizes the frequency-filtering effect of the intervening tissues. Under the linear time-invariant (LTI) assumption, where tissue dielectric properties depend only on frequency within the safe current range and the electrode-nerve geometry is fixed, we transformed arbitrary input waveforms *x*(*t*) via FFT, multiplied the spectrum by *H*(*f*), and reconstructed the time-domain response *y*(*t*) by inverse FFT. The root-mean-square (RMS) value of *y*(*t*) was taken as a quantitative measure of stimulation intensity. This framework was validated by reproducing the experimental observations of Hsu et al. [7], and the effect of electrode spatial arrangement on depth selectivity was examined through 25 configurations of a 7 × 6 electrode array. In addition, we introduce a “penetration ratio” to quantify the relative activation of deep versus superficial nerves across different waveforms and electrode configurations.

Regarding the choice of neural activation metric, more accurate predictions can be achieved through multiscale modeling: extracellular potentials obtained from finite element simulation can be imported into axon dynamics software such as NEURON to solve nonlinear membrane equations and simulate the actual generation of action potentials [22,23]. However, our objective is to compare relative activation strengths across different waveforms rather than to predict absolute firing thresholds. The activating function is therefore appropriate as a linear predictor for this purpose, since it has been widely validated for predicting neural depolarization under extracellular stimulation and preserves the relative ranking of waveforms under fixed electrode-nerve geometry.

## 2. Methods

The overall workflow consisted of three stages: geometric modeling in UGNX 2206, finite element simulation in COMSOL Multiphysics 6.1, and signal analysis using a custom Python program (Python 3.12). The frequency-dependent dielectric properties of all tissues were incorporated as described in Section 2.3. The activating function for each nerve, which served as the transfer function, was obtained from the frequency-domain simulation. Based on this transfer function, the Python program computed the time-domain neural response to arbitrary stimulation waveforms.

### 2.1. Geometric Model Construction

**Figure 1:**
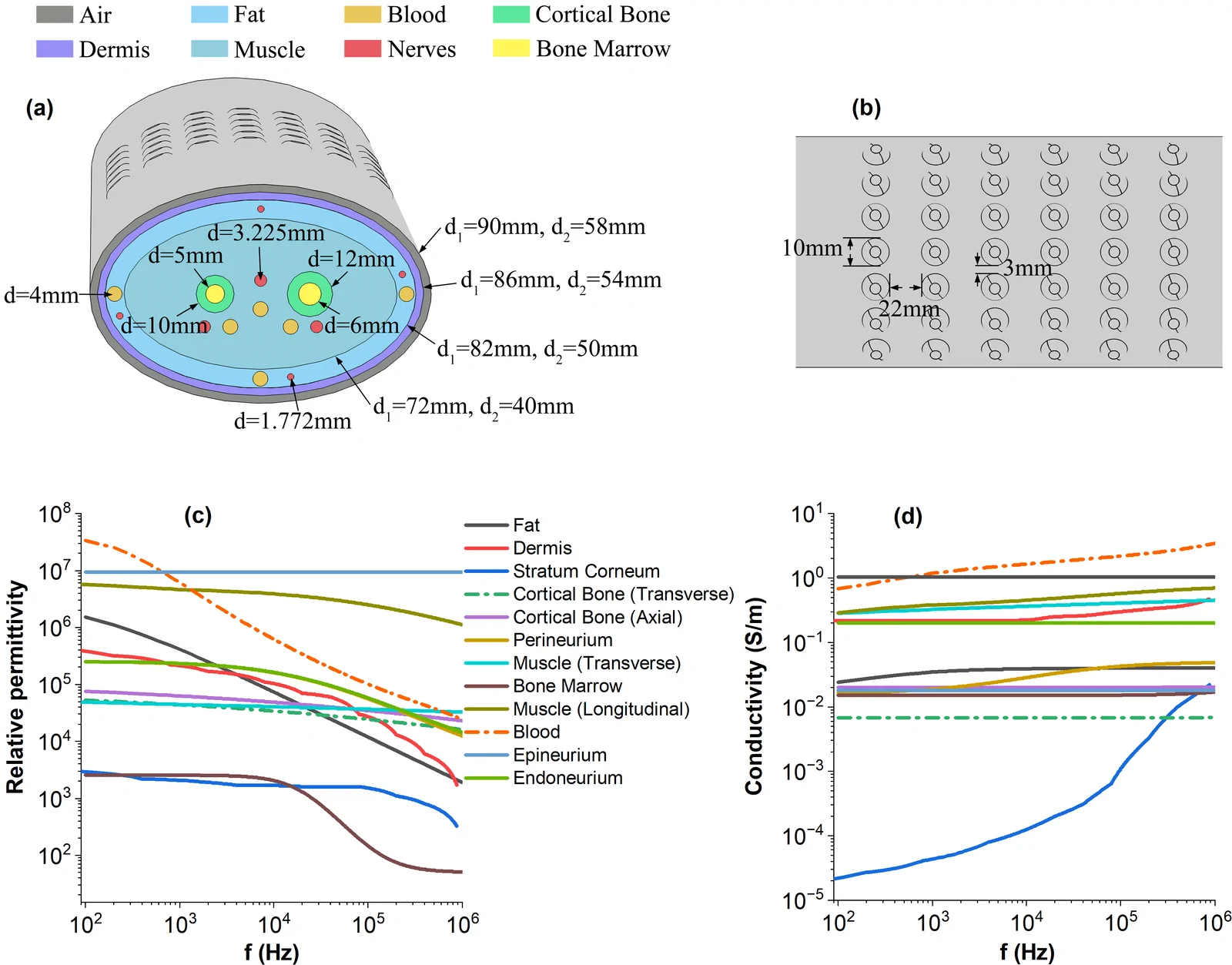
Research tools, forearm model, and tissue electrical properties. (**a**) The finite element geometric model of the forearm with multilayer tissue and the key dimensions. A 7 × 6 electrode array is shown on one surface of the model. (**b**) Front view of the electrode array, indicating electrode diameter and transverse/longitudinal spacing. (**c**,**d**) Frequency-dependent curves of (**c**) conductivity and (**d**) relative permittivity for major tissues (skin, fat, muscle, etc.) from 1 Hz to 1 MHz.

**Figure 2:**
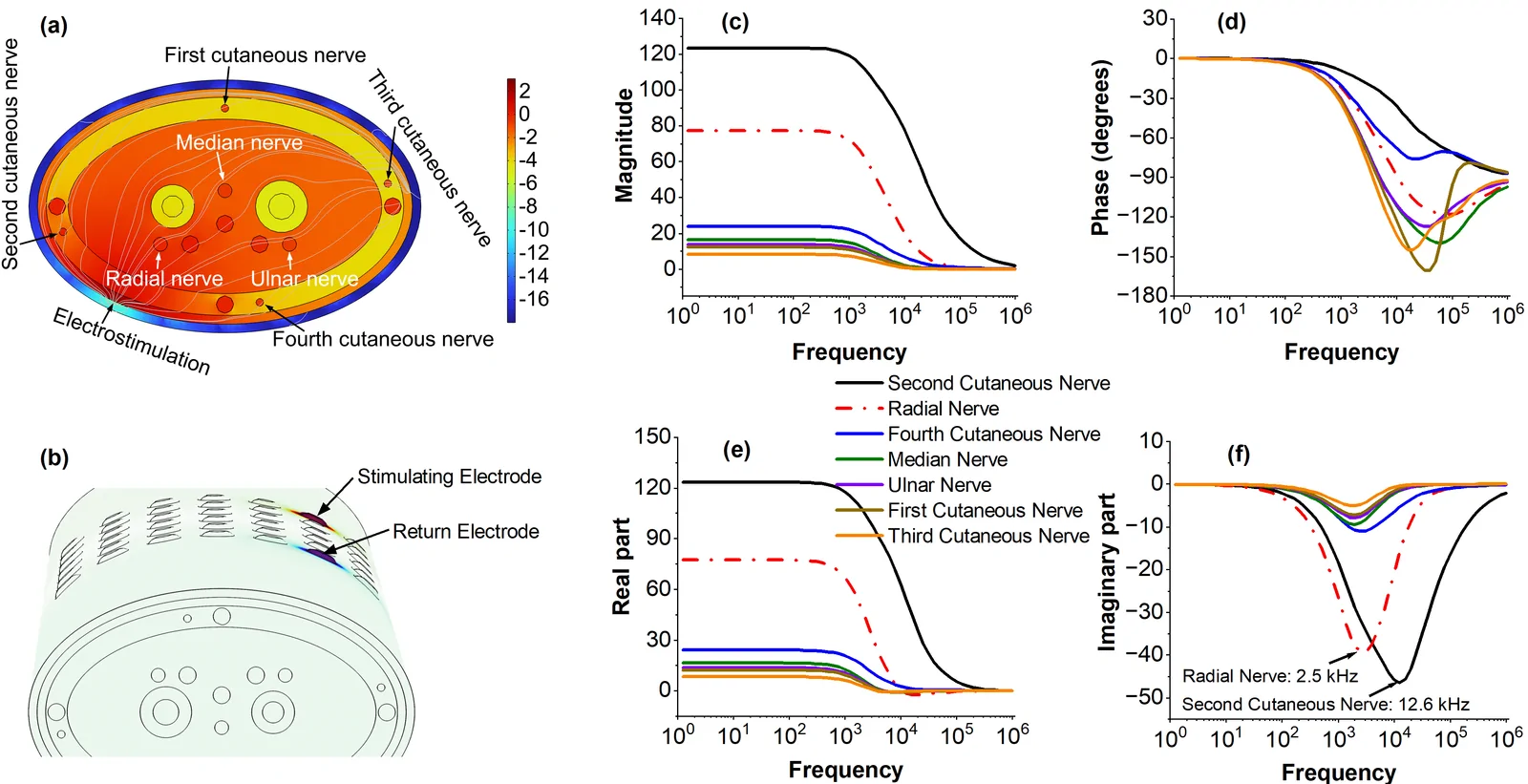
(**a**) Cross-sectional map of current-density magnitude (log_10_-transformed, units: A/m^2^) and flow direction in tissues, with positions of seven target nerves labeled. (**b**) Schematic of the electrode configuration used for the subsequent waveform-dependent neural activation study. The active electrode is positioned close to the Radial nerve and away from the cutaneous nerves. (**c**–**f**) Frequency response functions for the seven nerves from 0 Hz to 1 MHz: (**c**) magnitude frequency response, (**d**) phase frequency response, (**e**) real part, (**f**) imaginary part. The legend order corresponds to the magnitude level at low frequency.

A simplified multi-layer cylindrical model of the adult male forearm was constructed (Figure 1a). From outermost to innermost, the model comprised skin (stratum corneum and dermis), subcutaneous fat, muscle, cortical bone, and bone marrow. Representative blood vessels and nerves crossing the subcutaneous fat and muscle layers were embedded (Figure 2a). This simplification is justified because the electric field distribution is primarily governed by conductivity contrasts between tissue layers rather than by detailed boundary geometries.

Nerves were represented as three-layer coaxial cylinders: endoneurium, perineurium, and epineurium. The diameters of cutaneous and muscle nerves were 2 mm and 4 mm, respectively. For cutaneous nerves, the nerve fascicle occupies 90% of the total nerve diameter, i.e., 1.8 mm; for muscle nerves, it occupies 81.25%, i.e., 3.25 mm. The perineurium thickness was constant at 0.014 mm [24]. Therefore, the endoneurial diameter of the cutaneous nerve is $d_{end}=1.8-2\times 0.014=1.772\;mm$ (Figure 1a); the epineurium thickness of the cutaneous nerve is $d_{epi}=\;\left(2-2*0.014-1.772\right)/2=0.1\;mm$. Similarly, for the muscle nerve, the endoneurial diameter is 3.225 mm, and the epineurium thickness is 0.374 mm. In geometric modeling, the perineurium and epineurium were not constructed as independent geometries but were assigned electrical properties via the “Layered Material Stack” feature.

To accurately simulate the high impedance of the stratum corneum and avoid meshing difficulties due to its extreme thinness, a 4 mm thick air domain was added outside the dermis. At the air-dermis and silver electrode–dermis interfaces, the “Contact Impedance” boundary condition was used to model the electrical effect of the stratum corneum equivalent to a 20 μm thickness. A 7 × 6 rectangular silver electrode array was placed on the dorsal and ventral surfaces of the forearm model (Figure 1b) to simulate multi-channel surface stimulation scenarios.

### 2.2. Physics Settings and Mesh

This study employed the “Electric Currents (ec)” physics interface within the AC/DC module of COMSOL Multiphysics. A “Frequency Domain” study was performed using a direct solver, with a frequency sweep from 0 Hz to 1 MHz to incorporate the dielectric dispersion of the tissues. Under the quasi-static approximation, the governing equations solved by this interface are

(1) $$\nabla \cdot \left(\sigma +j\omega \epsilon \right)\nabla \phi =0,$$

(2) E=−∇ϕ.

Here, *σ* is tissue conductivity (S/m), ϵ is permittivity (F/m); ω = 2πf is angular frequency, and f is frequency (Hz); ϕ and **E** are electric potential (V) and electric field strength (V/m), respectively. On the designated active electrode, a “Terminal” boundary condition with a unit current excitation of 1 mA was applied to compute the system transfer function. On the end faces of the cylindrical model, a “Floating Potential” boundary condition was used to allow current to exit the domain. All remaining external surfaces (i.e., the outer boundary of the air domain) were set to “Electric Insulation”.

Mesh generation was performed as follows. One end face of the cylinder was discretized using triangular elements. From that face, a swept mesh was extruded along the axial direction. In regions containing electrodes, the geometry was irregular and did not allow a swept mesh; therefore, free tetrahedral meshing was used in those remaining parts.

### 2.3. Dielectric Properties of Tissues

The dielectric properties of all biological tissues were set as functions of frequency (Figure 1c,d). The conductivity and relative permittivity data for skin (stratum corneum and dermis) were sourced from Miklavčič et al. [25]. Other tissues were described using Cole–Cole relaxation models or derived formulas. Subcutaneous fat parameters were based on Gabriel et al. [9,10,26]:

(3) $$\sigma_{fat}\left(f\right)=0.04+\frac{0.015-0.04}{1+{\left(f/200\right)}^{0.8}}$$

(4) $$\epsilon_{r,fat}\left(f\right)=5+\frac{3\times 10^{6}}{1+{\left(f/100\right)}^{0.8}}$$

Muscle tissue is anisotropic [9,27,28], with axial (parallel to muscle fibers, ∥) and transverse (perpendicular to muscle fibers, ⊥) conductivity and relative permittivity given by

(5) $$\sigma_{mus,∥}\left(f\right)=0.8-\frac{0.45}{1+{\left(2\pi f\times 1.59\times 10^{-6}\right)}^{0.54}}$$

(6) $$\epsilon_{r,mus,∥}\left(f\right)=40+\frac{5\times 10^{6}-40}{1+{\left(2\pi f\times 1.59\times 10^{-6}\right)}^{0.54}}$$

(7) $$\sigma_{mus,⊥}\left(f\right)=0.8-\frac{0.75}{1+{\left(2\pi f\times 6.37\times 10^{-7}\right)}^{0.1}}$$

(8) $$\epsilon_{r,mus,⊥}\left(f\right)=40+\frac{7\times 10^{4}-40}{1+{\left(2\pi f\times 6.37\times 10^{-7}\right)}^{0.1}}$$

Cortical bone also exhibits marked electrical anisotropy. The longitudinal conductivity is three times the transverse conductivity [29]; the transverse relative permittivity is 70% of the longitudinal relative permittivity [9]:

(9) $$\sigma_{cor,∥}\left(f\right)=0.02\times \left(1+2\times 10^{-8}f\right)$$

(10) $$\sigma_{cor,⊥}\left(f\right)=0.0067\times \left(1+2\times 10^{-8}f\right)$$

(11) $$\epsilon_{r,cor,∥}\left(f\right)=15+\frac{1\times 10^{5}-15}{1+{\left(f/8000\right)}^{0.25}}$$

(12) $$\epsilon_{r,cor,⊥}\left(f\right)=10.5+\frac{7\times 10^{4}-10.5}{1+{\left(f/8000\right)}^{0.25}}$$

Bone marrow was treated as isotropic:

(13) $$\sigma_{mar}\left(f\right)=0.015+2\times 10^{-8}f$$

(14) $$\epsilon_{r,mar}\left(f\right)=50+\frac{2500}{1+{\left(f/20000\right)}^{2}}+\left(f=0\right)\times 3000$$

The electrical properties of the three nerve layers were based on data from Horn et al. [24]. The epineurium was treated as a purely resistive layer with conductivity and relative permittivity of 0.018 S/m and 9.4 × 10^−6^, respectively. The perineurium and endoneurium used frequency-dependent models:

(15) $$\sigma_{per}\left(f\right)=0.05+\frac{0.016-0.05}{1+{\left(2\pi f\cdot 8\times 10^{-6}\right)}^{0.8}}$$

(16) $$\epsilon_{r,per}\left(f\right)=2000+\frac{2.5\times 10^{5}}{1+{\left(2\pi f\cdot 8\times 10^{-6}\right)}^{0.8}}$$

The conductivity of endoneurium is anisotropic, with a transverse conductivity of 0.2 S/m and a longitudinal conductivity of 1.03 S/m. Relative permittivity is

(17) $$\epsilon_{r,end}\left(f\right)=4000+\frac{2.5\times 10^{5}}{1+{\left(2\pi f\cdot 8\times 10^{-6}\right)}^{0.8}}$$

The dielectric response of blood was described by a three-dispersion Cole–Cole model [30], with its complex dielectric spectrum being a superposition of α, β, and γ dispersions [10]:

(18) $$\sigma_{blood}\left(f\right)=0.6+\omega \epsilon_{0}\sum_{i=1}^{3}∆\epsilon_{i}\cdot \frac{{\left(\omega \tau_{i}\right)}^{1-\alpha_{i}}cos\left(\alpha_{i}\pi =/2\right)}{1+2{\left(\omega \tau_{i}\right)}^{1-\alpha_{i}}sin\left(\alpha_{i}\pi /2\right)+{\left(\omega \tau_{i}\right)}^{2\left(1-\alpha_{i}\right)}}$$

(19) $$\epsilon_{r,blood}\left(f\right)=4+\sum_{i=1}^{3}∆\epsilon_{i}\cdot \frac{1+{\left(\omega \tau_{i}\right)}^{1-\alpha_{i}}sin\left(\alpha_{i}\pi /2\right)}{1+2{\left(\omega \tau_{i}\right)}^{1-\alpha_{i}}sin\left(\alpha_{i}\pi /2\right)+{\left(\omega \tau_{i}\right)}^{2\left(1-\alpha_{i}\right)}}$$

where $\epsilon_{\infty }=4$, $\epsilon_{0}=8.854\times 10^{-12}$ F/m. Other parameters are shown in Table 1. The air layer was assigned a conductivity of 5 × 10^−13^ S/m and a relative permittivity of 1, while the silver electrode was given a conductivity of 61.6 × 10^6^ S/m and a relative permittivity of 1. The frequency-dependent conductivity and relative permittivity of all tissues are plotted in Figure 1c,d, and their complete material property parameters are summarized in Table S1 (Supporting Information).

### 2.4. Waveform Processing and Experimental Reproduction

To quantitatively compare the waveforms used in the psychophysical experiments of Hsu et al. [7], an automated Python analysis workflow was developed based on linear time-invariant system theory. First, the system transfer function *H*(*f*) was computed in COMSOL using the frequency-domain study (0–1 MHz). It was defined as the ratio of the second spatial derivative of the electric potential along the nerve axis (the activating function) at a fixed observation point to the input current:

(20) $$H\left(f,z_{0}\right)=\frac{\partial^{2}V(f,\;z_{0})/{\partial z}^{2}}{I_{in}\left(f\right)}$$

where z_0_ denotes the position at the center of the nerve directly beneath the circular stimulating electrode, where the electric field gradient is maximal. Under the unit’s current excitation of 1 mA, $H\left(f\right)$ equals the activating function numerically.

Subsequently, the program generated a waveform library including, but not limited to, all waveform types in Hsu’s study [7]. For any input time-domain waveform *x*(*t*), its frequency-domain representation *X*(*f*) was obtained via Fast Fourier Transform (FFT). The FFT was implemented using NumPy’s fft function (Cooley–Tukey algorithm). For each waveform, the number of FFT points *N* was set equal to the number of samples in *x*(*t*), which ranged from 100 to 1,000,000 depending on the waveform duration and sampling rate (for amplitude-modulated waves, the upper limit was 2,000,000). The sampling interval Δ*t* was determined by the time vector generated for each waveform, with the sampling rate of *f_s_* = 1/Δ*t* automatically adjusted to satisfy the Nyquist criterion: *f_s_* ≥ 2.4 × *f_max_*, where the 20% safety margin accounts for high-frequency harmonics in non-sinusoidal waveforms. *f_max_* is the highest significant frequency component of the waveform (e.g., for sine waves, *f_max_* = carrier frequency; for square waves, *f_max_* = 15 × fundamental frequency; for rectangular waves, *f_max_* = 5 × fundamental frequency). The frequency resolution was Δ*f* = *f_s_*/*N*. No additional window function was applied (i.e., a rectangular window), as the waveforms were generated with an integer number of cycles, minimizing spectral leakage. The inverse FFT used the same number of points *N*.

The frequency-domain system response *Y*(*f*) was the product of the transfer function and the input spectrum:

(21) Y(f)=H(f)⋅X(f)

The time-domain neural response *y*(*t*), which incorporates frequency-dependent tissue properties, was reconstructed by applying the inverse FFT to *Y*(*f*). Finally, the root-mean-square (RMS) value of *y*(*t*) was computed as a quantitative measure of “stimulation intensity” for each waveform:

(22) $$y_{rms}=\sqrt{\frac{1}{T}\int_{0}^{T}{\left\lfloor y\left(t\right)\right\rfloor }^{2}dt}$$

## 3. Results and Discussion

### 3.1. Frequency Response Characteristics of Nerves

The current density distribution within the tissues is shown in Figure 2a. Current flows primarily through pathways with higher conductivity, namely muscle and the dermis (muscle > dermis > fat, as shown in Figure 1c). The representative nerves are labeled in order of increasing geometric distance from the stimulating electrode: radial nerve < second cutaneous nerve < fourth cutaneous nerve < ulnar/median nerve < first cutaneous nerve < third cutaneous nerve. Figure 2c–f present the frequency response of the transfer function (magnitude, phase, real part, and imaginary part) for all nerves from 0 Hz to 1 MHz. These real and imaginary parts are steady-state frequency-domain quantities obtained from the sinusoidal steady-state solution; they are independent of the time origin and uniquely characterize the linear system’s response at each frequency.

Magnitude response (Figure 2c), defined as $|H(f)|=\sqrt{{Re}^{2}+{Im}^{2}}$, represents the stimulation intensity perceived by the nerve. The magnitude for all nerves decays approximately exponentially with increasing frequency. This indicates that, although tissue conductivity increases somewhat with frequency (Figure 1c), the stimulation intensity sensed by the nerves is predominantly governed by the sharp decline in permittivity (Figure 1d). The magnitude is generally negatively correlated with the nerve-electrode distance. However, the response magnitude of the radial nerve is lower than that of the more distant second cutaneous nerve. This anomaly is caused by the current reaching the deeper radial nerve having to traverse the fat–muscle interface, which exhibits a significant contrast in conductivity. The strong polarization effect at this interface creates an additional layer of capacitive impedance, thereby substantially attenuating the effective stimulating field strength.

Phase response (Figure 2d), defined as arctan (Im/Re), reflects the lag of the neural response relative to the driving electric field. The phase for all nerves starts from a value near 0° at low frequencies and asymptotically approaches −90° at high frequencies, indicating that at the high-frequency limit, all pathways are dominated by the displacement current mechanism (capacitive coupling). The approach to this limit exhibits three distinct patterns. First, the phase of the second cutaneous nerve decreases monotonically, behaving similarly to a simple first-order RC low-pass filter. Second, the phase curves for the radial, ulnar, median, and third cutaneous nerves show a non-monotonic characteristic: an initial decrease, passing through a trough, followed by a recovery. This behavior occurs because, compared with the path to the second cutaneous nerve, the current paths to these nerves must additionally cross the fat–muscle interface (a region of significant conductivity contrast) and then flow through muscle tissue. Their system response thus more closely resembles a distributed-parameter circuit model with multiple relaxation time constants. The response pattern of the third cutaneous nerve resembles that of the muscle nerves because its current path also primarily traverses muscle (Figure 2a). Third, the phase curves of the first and fourth cutaneous nerves exhibit two local extrema during their descent. This is caused by the existence of parallel pathways for the current reaching these nerves: one component travels through muscle and another through subcutaneous fat. These parallel branches possess different characteristic relaxation frequencies, resulting in the double extrema observed in the phase curve. Closer inspection reveals a subtle similarity in the phase response of the third cutaneous nerve to that of the first and fourth nerves (two faint extrema), which is attributable to partial similarity in their current paths, i.e., partially through fat but predominantly through muscle (Figure 2a).

The real part of the response reflects the component of the neural response that is in phase with the stimulus signal. As shown in Figure 2e, the real part decays with increasing frequency, gradually approaching zero from an initial positive value. This confirms the dominant role of decreasing permittivity. Notably, unlike the monotonic decay observed for the second and fourth cutaneous nerves, the real-part curves for the remaining nerves all display a transient negative region. This is attributed to their current paths encompassing the fat–muscle interface with its significant conductivity contrast (Figure 2a). The pronounced dielectric relaxation effect at this interface causes the induced electric field vector at the nerve location to transiently oppose the direction of the original driving field, leading to a negative real-part response.

The imaginary-part response reflects the component of the neural response that is orthogonal (90° out of phase) to the stimulus signal. As shown in Figure 2f, the imaginary part starts from a small negative value near zero, decreases with frequency to a trough, then rises again and asymptotically approaches zero. This variation reflects the system transitioning from capacitive dominance at low frequencies, passing through a point of maximum energy dissipation, and gradually returning to resistive dominance. The trough of the curve corresponds to the most prominent characteristic dielectric relaxation frequency of the specific neural pathway, where energy dissipation due to polarization lag is maximized. For neural stimulation, signals near this trough frequency should be avoided to minimize unnecessary energy dissipation.

In summary, the frequency response characteristics of a nerve are jointly determined by its spatial geometric relationship with the stimulating electrodes and the conductive properties of the tissues along the current path. First, the geometric distance between the nerve and the electrode, combined with the static conductivity of the intervening tissues, dictates the overall magnitude (i.e., stimulation intensity) of the nerve’s response function. Second, the frequency-dependent complex impedance properties of the tissues encountered by the current path modulate the phase-frequency characteristics and the complex variation patterns of the real and imaginary parts.

### 3.2. Experimental Validation

To validate the simulation model, we compared our results with the five experiments carried out by Hsu et al. [7].

**Figure 3:**
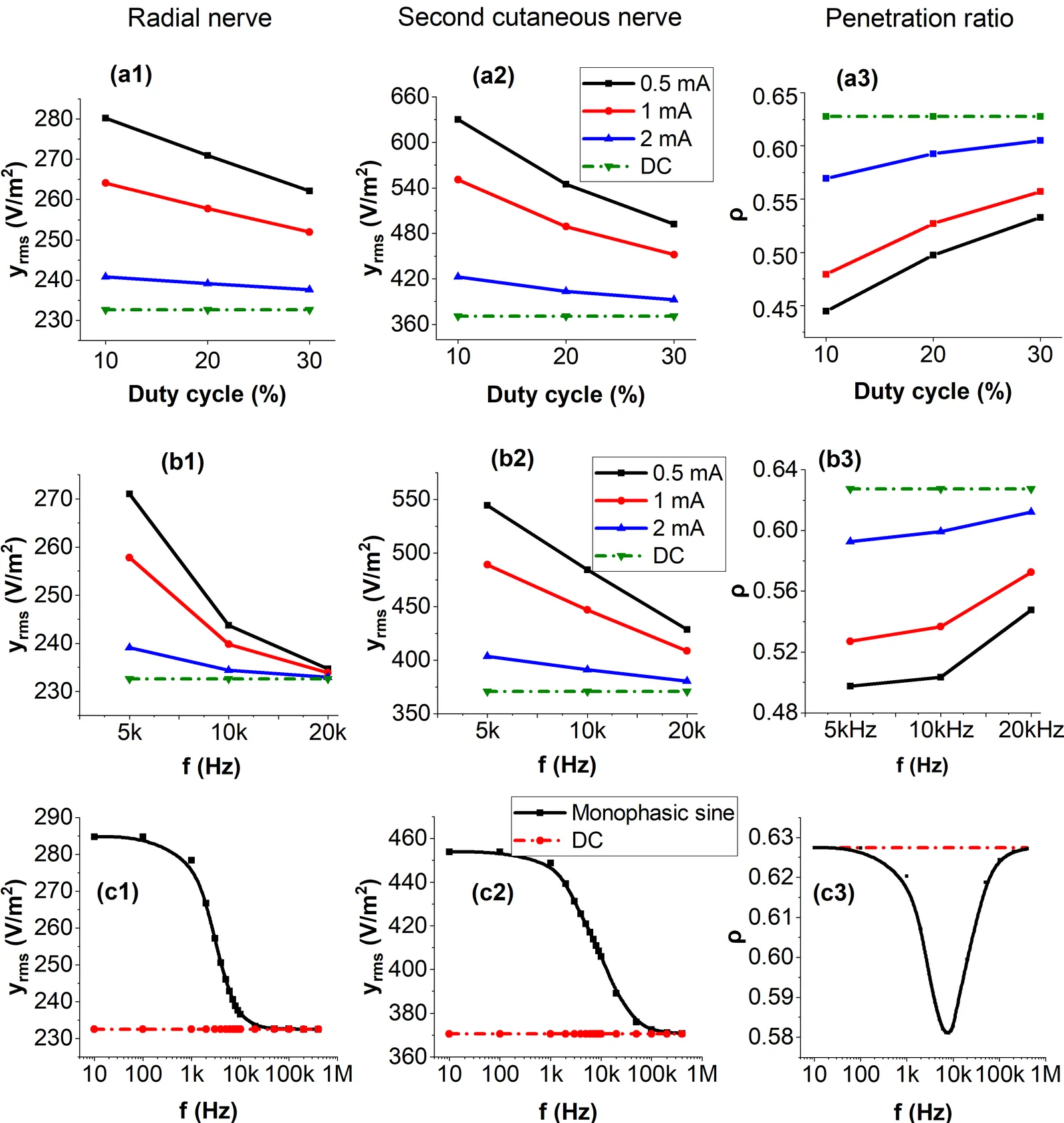
Effects of monophasic waveform parameters on stimulation intensity. (**a1**–**a3**) Duty cycle and baseline current effects at 5 kHz: (**a1**) RMS stimulation intensity for the radial nerve; (**a2**) for the second cutaneous nerve; (**a3**) penetration ratio ρ = y_Radial/_y_Second_cutaneous_. (**b1**–**b3**) Frequency dependence at 20% duty cycle: (**b1**) radial nerve; (**b2**) second cutaneous nerve; (**b3**) ρ. (**c1**–**c3**) Monophasic sine waves: (**c1**) radial nerve; (**c2**) second cutaneous nerve; (**c3**) ρ.

**Experiment 1: Monophasic square waves—duty cycle and baseline current.** Hsu et al. tested 5 kHz monophasic square waves with duty cycles of 10%, 20%, and 30% and baseline currents of 0.5, 1, and 2 mA, all delivering a net current of 3 mA [7]. They found that sensation intensity decreased significantly with increasing duty cycle or baseline current but never fell below the level of constant direct current (DC). Our simulations reproduced this trend exactly: as shown in Figure 3(a1,a2), the calculated stimulation intensity (y_rms_) for both the deep radial nerve and the superficial second cutaneous nerve decreased monotonically with a higher duty cycle or higher baseline current, while remaining above the DC level. This implies that any adjustment that reduces superficial sensation by increasing duty cycle or increasing baseline current inevitably reduces the absolute stimulation intensity for deep muscle nerves. This trade-off is consistent with previous studies showing that stimulating deep nerves unavoidably co-activates superficial nerves [21,31,32].

**Figure 4:**
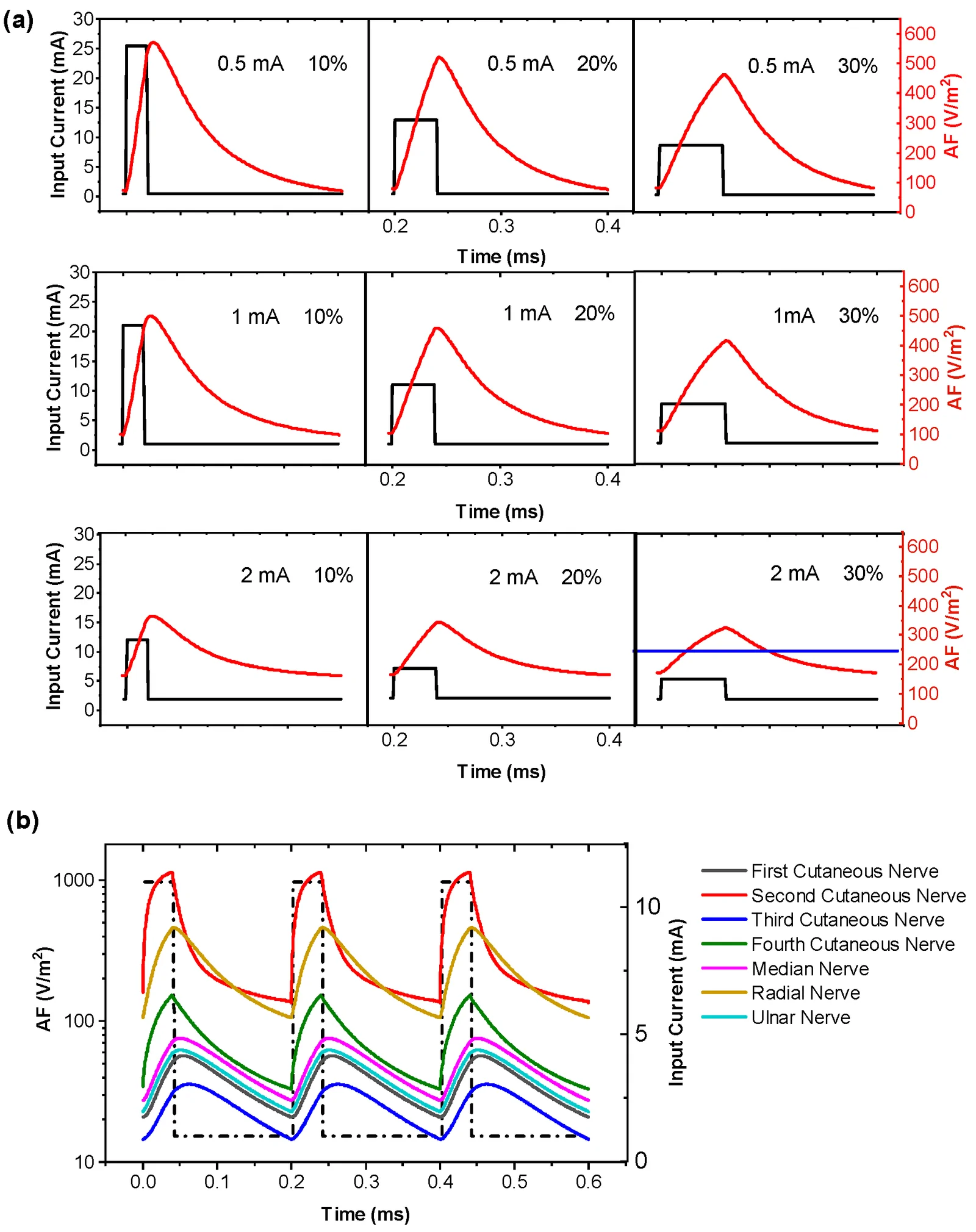
(**a**) Time-domain input current waveforms (black) and the corresponding neural responses on the radial nerve (red) for all monophasic square wave conditions tested in Experiment 1 of Hsu et al. [7]. The blue horizontal line (bottom right) represents the response to 3 mA DC stimulation. (**b**) Time-domain activating function responses for the seven nerves under a representative monophasic square wave condition (5 kHz, 20% duty cycle).

Figure 4 displays the time-domain input current waveforms and the corresponding neural responses. The response to square wave stimulation exhibits an approximately triangular shape. This shape arises because the tissue acts as a low-pass filter: the high-frequency components of the square wave are attenuated, and the membrane capacitance integrates the abrupt transitions, producing a near-linear rise and fall of the activating function. The peak amplitude decreases with increasing duty cycle or baseline current but always remains above the constant DC level. This aligns with the conclusion of Hsu et al. that any signal fluctuation deviating from a constant current induces a stronger neural sensation [7]. They attributed this phenomenon to the greater propensity of abrupt signal transitions (e.g., rising edges) to activate nerve fibers. The present study provides an electrical-level explanation based on the quasi-static field distribution.

For a representative monophasic square wave condition (5 kHz, 20% duty cycle), Figure 4b shows the time-domain activating function responses for all seven nerves. The response magnitude generally follows the nerve-electrode distance: nerves closer to the stimulation site exhibit larger activating function amplitudes, while those farther away show progressively smaller responses. In addition, a clear delay is observed for nerves farther from the electrode.

To evaluate depth selectivity independently of absolute intensity, we defined the penetration ratio as ρ = y_Radial_/_Second_cutaneous_. As shown in Figure 3(a3), ρ increased with duty cycle or baseline current, indicating that these parameters improve the relative activation efficiency of deep nerves. However, ρ for all monophasic square waves remained below that of DC, meaning that no pulsed waveform achieved better depth penetration than constant current.

**Experiment 2: Monophasic square waves—frequency dependence.** Hsu et al. varied the frequency of 20% duty-cycle monophasic square waves (5, 10, 20 kHz) while keeping the net current at 3 mA [7]. They reported that sensation decreased with increasing frequency. Our simulations confirmed this: as shown in Figure 3(b1,b2), the stimulation intensity (y_rms_) for both the deep radial nerve and the superficial second cutaneous nerve decreased with frequency while remaining above the DC level.

As frequency increased, the time-domain response waveform gradually transitioned from a triangular shape toward a sinusoidal profile (Supplementary Figure S1). This transition reflects the low-pass filtering effect of the biological tissues, which attenuate high-frequency components more strongly.

As shown in Figure 3(b3), the penetration ratio ρ also increased with frequency, indicating that higher frequencies improve the relative activation efficiency of deep nerves, but it remained below that of DC in all cases.

**Experiment 3: Monophasic sine waves.** Hsu et al. applied monophasic sine waves with frequencies of 1, 5, 10, 25, and 50 kHz, each with 3 mA amplitude and a 3 mA DC offset (net current 3 mA, Figure S2), and compared them to constant DC [7]. They found that the sensation at 1 kHz was significantly higher than at all other frequencies (including DC), and participants reported a unique vibrating and itching sensation. Consequently, they did not explore lower frequencies. Our simulations reproduced this pattern: as shown in Figure 3(c1,c2), the stimulation intensity (y_rms_) for both nerves decayed exponentially with increasing frequency, approaching the DC level at high frequencies. The phase delay of the neural response relative to the stimulus also became progressively more pronounced at higher frequencies (Supplementary Figure S2).

Furthermore, studies on non-invasive electrical stimulation have reported that high-frequency (>1 kHz) carriers elicit weaker sensory responses, including temporal interference stimulation [31] and transcranial electrical stimulation [33,34]. Clinical studies of transcranial electrical stimulation have also found that, compared with constant direct current, oscillatory monophasic current stimulation is more likely to induce fatigue, with a minority of subjects reporting phosphenes and shaking of the visual field [35]. The present study provides an electrical mechanism explanation for these phenomena from a purely electrical perspective.

The penetration ratio ρ (Figure 3(c3)) exhibited a distinct trough at approximately 8 kHz, converging to the DC level at both low and high frequency ends. This trough can be explained by dielectric relaxation: as shown in Figure 2f, the characteristic relaxation frequencies of the radial and second cutaneous nerves are approximately 2.5 kHz and 12.6 kHz, respectively; their arithmetic mean (~7.55 kHz) closely matches the trough position. Thus, the frequency selection for monophasic sine waves should consider the dielectric relaxation properties of the intervening tissues.

**Figure 5:**
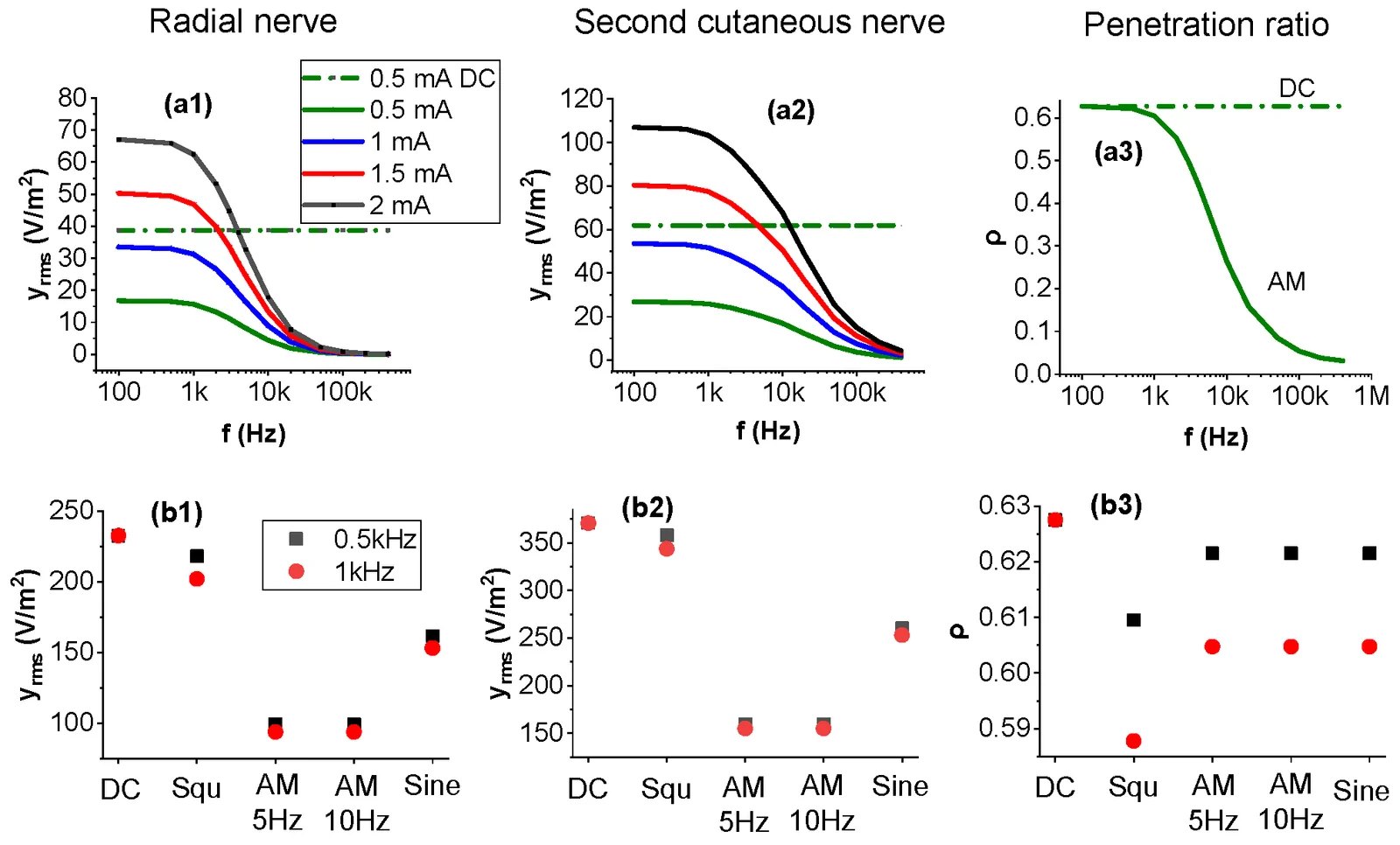
Stimulation characteristics of amplitude-modulated (AM) sine waves. (**a1**–**a3**) Effect of carrier frequency and amplitude at fixed modulation frequency (5 Hz): (**a1**) RMS stimulation intensity for the radial nerve; (**a2**) for the second cutaneous nerve; (**a3**) penetration ratio ρ = y_Radial/_y_Second_cutaneous_. (**b1**–**b3**) Comparison of different waveforms at fixed amplitude (3 mA): (**b1**) RMS stimulation intensity for the radial nerve; (**b2**) for the second cutaneous nerve; (**b3**) penetration ratio ρ. Waveforms compared: DC, square wave, sine wave, and AM waves with modulation frequencies of 5 Hz and 10 Hz.

**Experiment 4: Amplitude-modulated (AM) sine waves.** Hsu et al. tested biphasic AM waves with a fixed modulation frequency of 5 Hz, carrier frequencies of 100 Hz, 500 Hz, 1 kHz, and 2 kHz, and peak-to-peak amplitudes of 0.5, 1, 1.5, and 2 mA [7]. Figure S3 illustrates the time-domain shape of the AM waveforms used in this experiment. They reported that sensation decreased dramatically with increasing carrier frequency and increased with amplitude. Our simulations reproduced these monotonic trends and extended the carrier frequency range up to 400 kHz (Figure 5(a1,a2)). In addition, the stimulation intensity of the AM wave was lower than that of direct current (DC) under the condition of identical current amplitude.

As shown in Figure 5(a3), the penetration ratio ρ for AM waves decreased monotonically with increasing carrier frequency, in contrast to the non-monotonic behavior observed for monophasic sine waves (Figure 3(c3)). This difference arises because biphasic AM waves contain no DC component; their electric field distribution is shaped by capacitive coupling, which concentrates energy in superficial layers as carrier frequency increases.

**Experiment 5: Comparison of AC waveforms.** Hsu et al. compared square waves, sine waves, and AM waves (with modulation frequencies of 5 Hz and 10 Hz) at carrier frequencies of 500 Hz and 1 kHz, all with a maximum amplitude of 3 mA [7]. They found that sine waves produced the lowest sensation and square waves the highest. Our simulations agreed with the overall ordering: square waves consistently produced the highest stimulation intensity across both frequencies (Figure 5(b1,b2)).

However, a discrepancy was observed in the relative ranking of sine waves and AM waves. While Hsu et al. reported that at 1 kHz AM waves produced higher sensation than sine waves [7], our simulations indicated that sine waves consistently produced higher stimulation intensity than AM waves at both carrier frequencies (Figure 5(b1,b2)). The difference between 5 Hz and 10 Hz AM waves was also less pronounced in our model than in the experiment.

Under identical maximum amplitude (3 mA), all AC waveforms (square, sine, and AM) produced lower stimulation intensity than DC. This aligns with clinical evidence showing that DC-based electrical stimulation can be more effective than AC modalities for certain therapeutic applications [36]. In terms of depth selectivity, AM waves and sine waves performed similarly, both superior to square waves (Figure 5(b3)), yet all remained inferior to DC.

In addition to the experiments following the Hsu et al. protocol (with net current held constant), we performed a supplementary parametric study on monophasic square waves to isolate the effects of pulse width and frequency, with stimulus amplitude fixed at 1 mA. As shown in Supplementary Figure S4, the stimulation intensity (y_rms_) on the radial nerve increased monotonically with duty cycle (Figure S4(a1)). This is expected, as increasing duty cycle increases the time-averaged current injected into the tissue, thereby enhancing the overall stimulus strength. The corresponding penetration ratio (Figure S4(a2)) also increased monotonically with duty cycle. For the frequency dependence (Figure S4(b1)), the stimulation intensity decreased monotonically with increasing frequency, consistent with the low-pass filtering characteristics of the tissue. Notably, the penetration ratio as a function of frequency (Figure S4(b2)) exhibited a distinct trough at approximately 8 kHz, consistent with the trough observed for monophasic sine waves (Figure 3(c3)). This confirms that the frequency dependence of the penetration ratio is governed by the dielectric relaxation properties of the intervening tissues, rather than by the specific waveform shape.

Integrating the above experimental validations, three main findings emerge from the waveform analysis. First, under identical average current, monophasic waveforms produce higher stimulation intensity than DC due to their high-amplitude pulses; under identical peak-to-peak current, DC produces higher stimulation intensity than all biphasic waveforms (square, sine, and AM). Second, regardless of waveform type (monophasic or biphasic), the penetration ratio ρ remains below that of DC, demonstrating a universal depth-penetration advantage of constant direct current. This confirms a skin-effect-like behavior of electrical current in biological tissues, where time-varying currents concentrate in superficial layers. This behavior is attributed to dielectric relaxation at the fat–muscle interfaces, where capacitive shunting and the frequency-dependent decrease in tissue permittivity cause time-varying currents to concentrate in superficial layers. Third, any variation in waveform parameters that reduces superficial activation simultaneously decreases the stimulation intensity of deep muscle nerves.

### 3.3. Influence of Active Electrode Configuration

**Figure 6:**
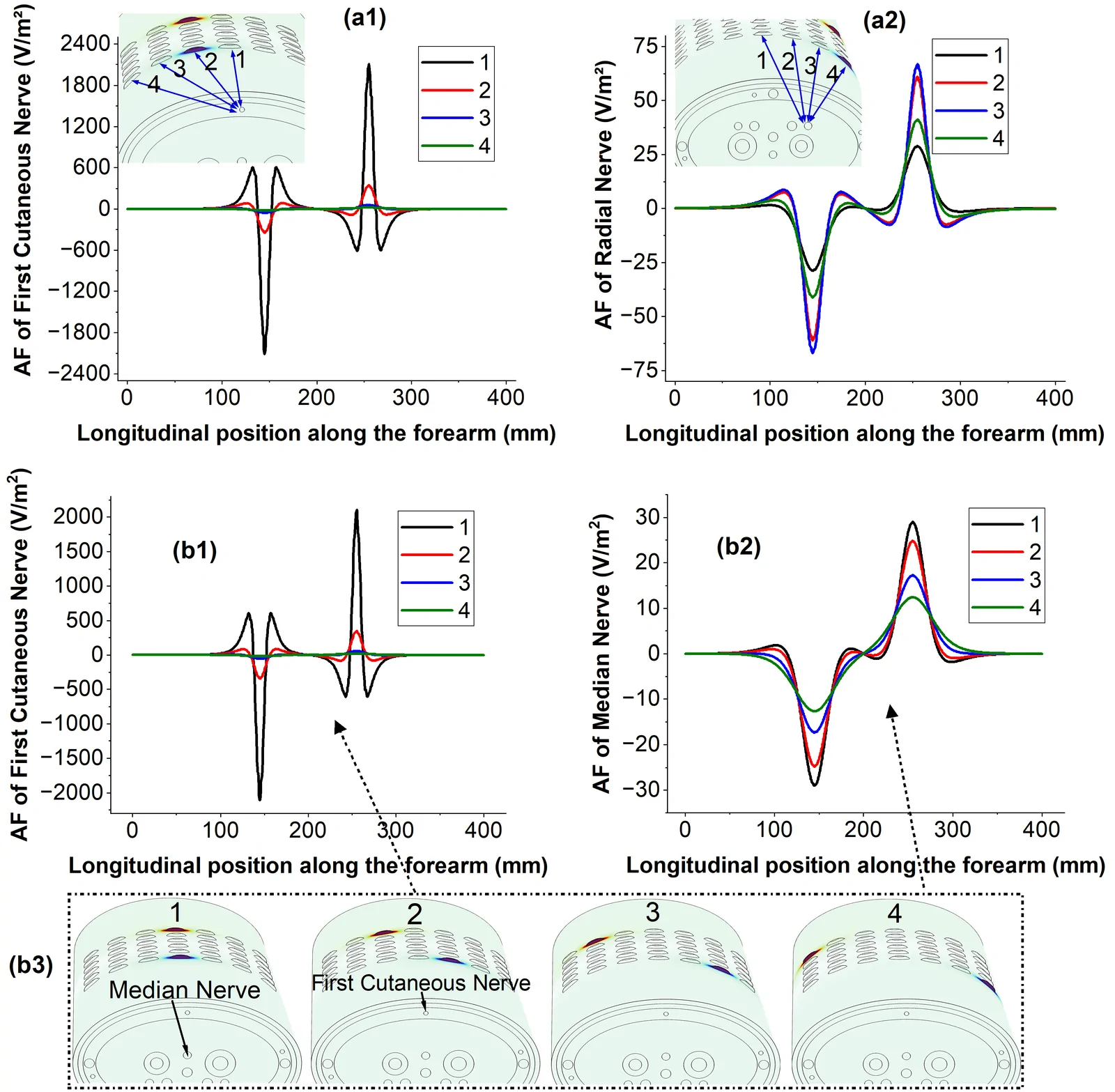
Effect of electrode-to-nerve distance on neural activation. (**a1**,**a2**) Magnitude of the activating function along the longitudinal axis of the forearm (0–400 mm): (**a1**) first cutaneous nerve; (**a2**) radial nerve. (**b1**,**b2**) Magnitude of the activating function as a function of transverse electrode spacing for (**b1**) the first cutaneous nerve and (**b2**) the target median nerve. (**b3**) Schematic diagrams of the electrode configurations corresponding to the transverse spacings in (**b1**,**b2**).

We first examined the effect of electrode-to-nerve distance. As shown in Figure 6(a1), the magnitude of the activating function on a cutaneous nerve (first cutaneous nerve) decreases rapidly as the electrode moves away from the nerve. Conversely, the activating function magnitude on a muscle nerve (radial nerve) increases when the electrode is placed closer to it (Figure 6(a2)). Thus, to minimize cutaneous discomfort, the active electrode should be positioned as far as possible from superficial cutaneous nerves and as close as possible to the target deep muscle nerve.

However, an anatomical conflict arises when a muscle nerve lies directly beneath a cutaneous nerve (e.g., the median nerve under the first cutaneous nerve, see Panel 3 in Figure 6(b3)). In such cases, placing the electrode near the deep target inevitably exposes the overlying cutaneous nerve to strong stimulation, causing pain.

To resolve this conflict, we increased the transverse distance between the active and return electrodes. This strategy effectively reduces the activating function magnitude on the first cutaneous nerve, whereas the reduction on the median nerve is much less pronounced (Figure 6(b1,b2)). With the configuration Panel 3 (Figure 6(b3)), the activating function magnitude on both nerves reaches a similarly low level. At this point, raising the total stimulation current can achieve effective deep activation while keeping superficial stimulation below the pain threshold.

**Figure 7:**
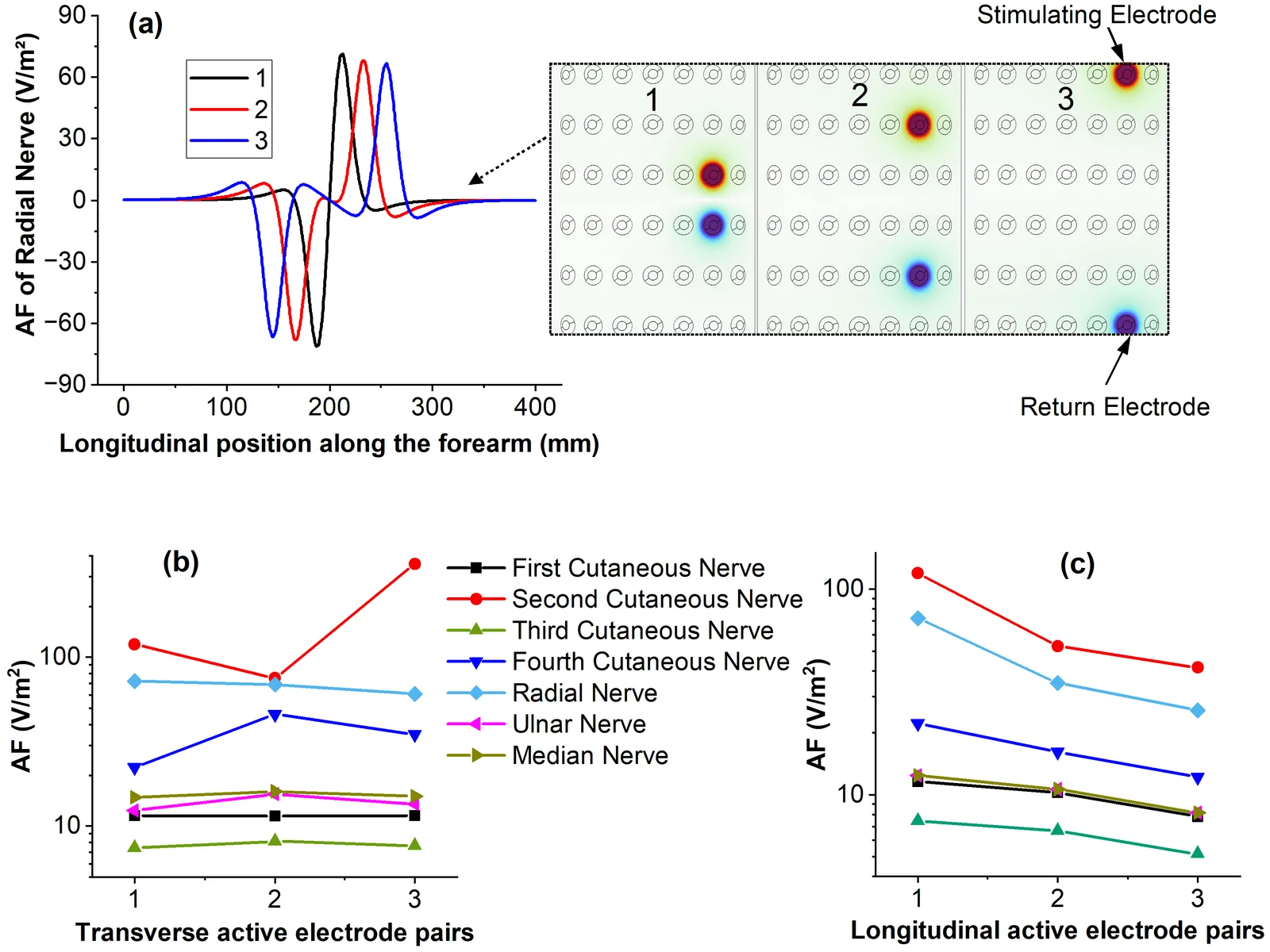
Effect of electrode-pair arrangement on neural activation. (**a**) Magnitude of the activating function on the target radial nerve along the longitudinal axis of the forearm (0–400 mm) for different longitudinal spacings between the active and return electrodes. (**b**) Peak magnitude of the activating function on each of the seven nerves as a function of the number of transversely arranged active electrode pairs (1, 2, or 3), with total current held constant. (**c**) Peak magnitude of the activating function on each of the seven nerves as a function of the number of longitudinally arranged active electrode pairs (1, 2, or 3), with total current held constant.

We then investigated the longitudinal spacing of the electrode pair at a favorable location near the radial nerve (Case 3 in Figure 6(a2)). As shown in Figure 7a, decreasing the longitudinal spacing causes only a slight, non-significant increase in the activating function magnitude. Because excessively small spacing may increase the risk of short-circuiting or current leakage [37], an optimal spacing that balances efficiency and safety should be determined in practice.

In addition to the position and spacing of a single electrode pair, we also examined stimulation scenarios with multi-electrode arrays. Under the condition of constant total input current, we increased the number of active electrodes in the transverse (perpendicular to the forearm) and longitudinal (parallel to the forearm) directions, respectively (Figure 7b,c).

**Figure 8:**
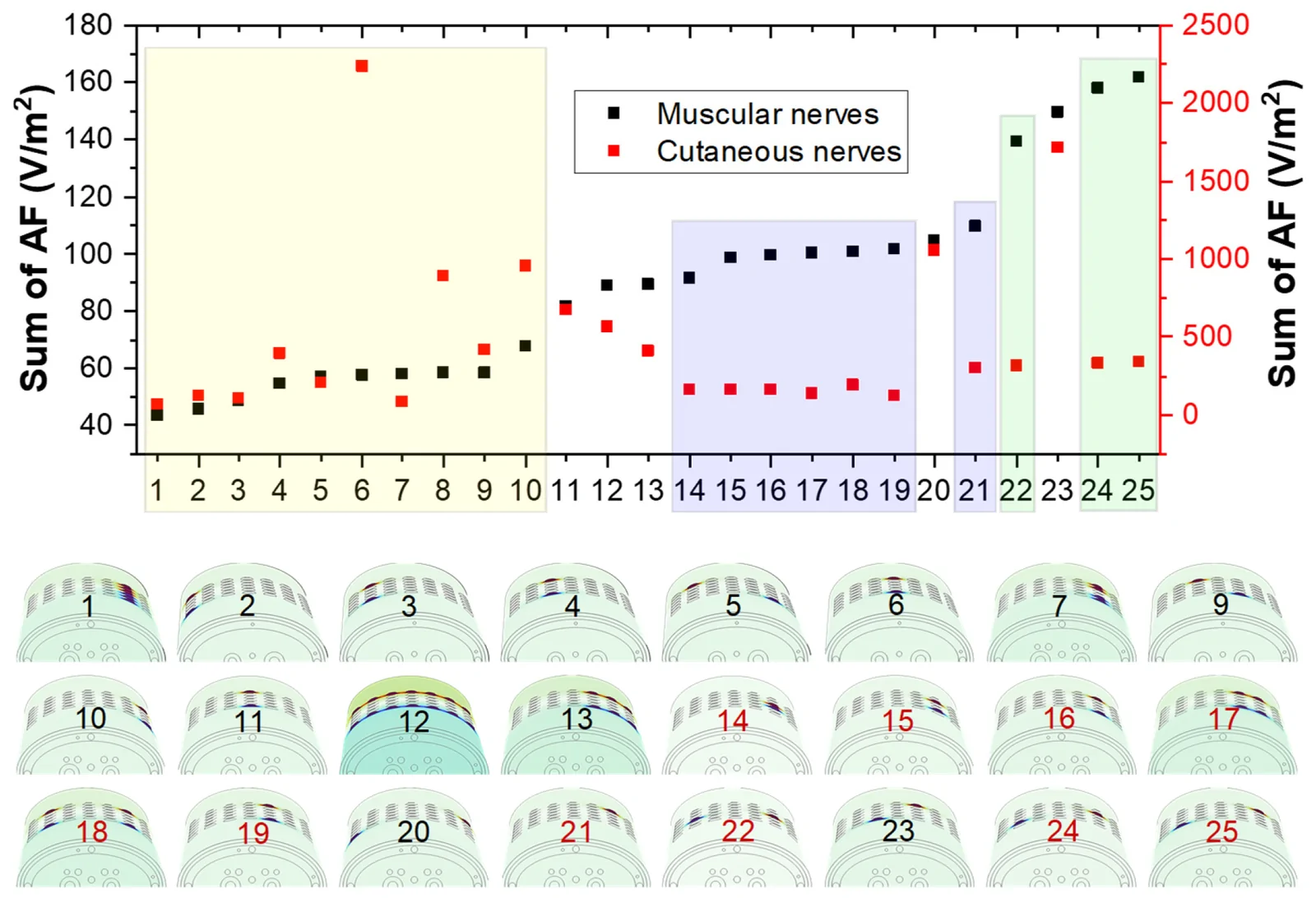
Comprehensive evaluation of neural-population activation across different electrode spatial configurations. Cumulative activating-function magnitude induced by 25 distinct electrode configurations (varying in position and spacing) is shown for all cutaneous nerves (red) and all muscle nerves (black). Model diagrams of 24 representative configurations are displayed below. The background colors indicate performance groups: yellow = worst, purple = acceptable, green = best. Note: Panel 8 shares similarities with Panel 5 and Panel 9, ranked by transverse electrode spacing as 9 < 5 < 8; in Panel 21, the return electrode is placed on the contralateral side of the forearm.

For transverse array expansion (Figure 7b), the effect was nerve dependent. For the second and fourth cutaneous nerves, which are anatomically closer to the added electrodes, the stimulation intensity with two or three electrode pairs was clearly higher than with a single pair (second cutaneous: 3 > 1 > 2; fourth cutaneous: 2 > 3 > 1). For the first and third cutaneous nerves, as well as for all muscle nerves, the differences across configurations were less pronounced. In fact, while the effect on individual muscle nerves was modest, transverse expansion enhanced the cumulative activation of all muscle nerves (see Figure 8), indicating that this strategy is effective for broader muscle nerve recruitment. However, electrode placement should avoid aligning the added electrodes with superficial cutaneous nerves, as this would increase unwanted cutaneous activation.

For longitudinal array expansion (Figure 7c), increasing the number of longitudinally arranged electrodes consistently reduced stimulation intensity across all seven nerves (1 > 2 > 3). This uniform decrease is attributed to the higher longitudinal conductivity of muscle tissue, which effectively increases the current-path cross-section when electrodes are extended longitudinally, thereby lowering current density and weakening stimulation regardless of nerve location. The detailed longitudinal distributions of the activating function for each nerve under the transverse and longitudinal array configurations are provided in Supplementary Figure S5.

To evaluate the overall effect of different electrode configurations on neural populations, we summed the activating function magnitude over all cutaneous nerves and over all muscle nerves for the 25 configurations (Figure 8). Schemes 1–10 (yellow background) are the worst performers. Schemes 2–5 and 8–10 have the active electrode far from the muscle nerves; Scheme 6 is particularly problematic, because the stimulating electrode is far from muscle nerves and simultaneously close to cutaneous nerves; and Schemes 1 and 7 have a large longitudinal contact area, which reduces current density. Schemes 11–13, although still near cutaneous nerves, show reduced stimulation of cutaneous nerves and increased stimulation of muscle nerves, because the electrode is shifted toward the muscle-aggregated side. Schemes 14–19 and 21 (purple background) are acceptable configurations; they place the electrode closer to the muscle side and farther from cutaneous nerves, further increasing muscle activation while decreasing cutaneous activation. Schemes 20 and 23 strongly stimulate both nerve types, because the electrode is close to both the muscle side and cutaneous nerves; these are also undesirable. Schemes 22, 24, and 25 (green background) best meet the ideal configuration criteria: they place the active electrode close to the muscle-nerve concentration and away from cutaneous nerves, while also increasing transverse spacing to expand coverage.

## 4. Conclusions

This study presents a computational framework for optimizing TES that addresses a key limitation of prior models: the neglect of frequency-dependent tissue dielectric properties. By incorporating frequency-dependent dielectric properties into a finite-element forearm model and coupling the results with a transfer-function-based signal-processing pipeline, we established a method capable of predicting the neural response to arbitrary stimulation waveforms. The quantitative reproduction of the psychophysical findings of Hsu et al. [7] validates this approach and confirms that frequency-dependent dielectric dispersion is a critical determinant of waveform-dependent neural activation.

Three principal findings emerge from the waveform analysis. First, under identical average current, monophasic waveforms produce higher stimulation intensity than DC; under identical peak current, DC produces higher stimulation intensity than all biphasic waveforms. Second, all time-varying waveforms exhibit a lower penetration ratio than DC, reflecting a skin-effect-like behavior in which time-varying currents preferentially concentrate in superficial tissue layers. Third, waveform parameter adjustments that reduce superficial sensory activation inevitably attenuate deep nerve activation, demonstrating that waveform optimization alone cannot resolve the depth selectivity problem.

Electrode spatial configuration offers a complementary strategy. The optimal configuration places the active electrode close to the target muscle nerve while maintaining distance from cutaneous nerves and employs a sufficiently large transverse span to reduce current density on superficial nerves.

Several limitations should be noted. The simplified cylindrical forearm model, while validated against experimental data, does not capture individual anatomical variations. The activating function, used as a linear proxy for neural activation, does not model the nonlinear dynamics of action potential initiation; however, because it depends linearly on the electric field and the transfer function is identical across waveforms under fixed geometry, the relative ranking of activation strengths is preserved, which suffices for the comparative objectives of this study.

In summary, this work demonstrates that the frequency-dependent dielectric properties of intervening tissues are a primary biophysical mechanism underlying waveform-dependent cutaneous sensation in TES. The transfer-function-based framework provides a computationally efficient tool for screening stimulation waveforms and electrode configurations and can be adapted to other anatomical targets.

## Figures and Tables

**Table 1:** Dielectric relaxation model parameters for blood.

Dispersion Type	$∆\varepsilon_{i}$	$\tau_{i}$	$\alpha_{i}$
α-dispersion	4.5 × 10^7^	6.8 × 10^−4^	0.17
β-dispersion	2.21 × 10^4^	1.33 × 10^−7^	0.12
γ-dispersion	87	8.33 × 10^−12^	0.07

## Data Availability

Data are contained within the article.

## Supplementary Materials

The following supporting information can be downloaded at: https://www.mdpi.com/article/10.3390/bioengineering13080847/s1.
