# Supplementary material for: Optimizing Transcutaneous Electrical Stimulation Based on Frequency-Dependent Tissue Modeling and Signal Analysis
Source: Bioengineering (Basel). 2026 Jul 23;13(8):847. doi: 10.3390/bioengineering13080847 (PMC13509627; doi:10.3390/bioengineering13080847)
Supplement: Supplementary file 1 [file bioengineering-13-00847-s001.zip › bioengineering-4394939-supplementary.pdf]

Optimizing Transcutaneous Electrical Stimulation Based on Frequency-Dependent Tissue Modeling and Signal Analysis

Table S1. Material properties of the finite element forearm model.

No.	Material	Role	Conductivity σ(f) [S/m]	Relative Permittivity $\varepsilon_r(f)$	Model Type	Ref.
1	Stratum corneum	Boundary (Contact Impedance, 20 μm)	Frequency-dependent data	Frequency-dependent data	Empirical	[13]
2	Dermis	Volume (t = 4 mm)	Frequency-dependent data	Frequency-dependent data	Empirical	[13]
3	Subcutaneous fat	Volume (t = 10 mm)	0.04 + (0.015 − 0.04) / (1 + (f / 200)^0.8)	$5 + 3\times10^6$ / (1 + (f / 100)^0.8)	Cole-Cole derived	[7,8,14]
4	Muscle (axial, ∥)	Volume (irregular)	0.8 − 0.45 / (1 + (2πf · 1.59×10^{-6})^0.54)	40 + (5×10^6 − 40) / (1 + (2πf · 1.59×10^{-6})^0.54)	Cole-Cole	[7,15,16]
5	Muscle (transverse, ⊥)	Volume (irregular)	0.8 − 0.75 / (1 + (2πf · 6.37×10^{-7})^0.1)	40 + (7×10^4 − 40) / (1 + (2πf · 6.37×10^{-7})^0.1)	Cole-Cole	[7,15,16]
6	Cortical bone (∥)	Volume (t = 6 mm)	0.02 × (1 + 2×10^{-8}f)	15 + (10^5 − 15) / (1 + (f / 8000)^0.25)	Empirical	[7,17]
7	Cortical bone (⊥)	Volume (t = 6 mm)	0.0067 × (1 + 2×10^{-8}f)	10.5 + (7×10^4 − 10.5) / (1 + (f / 8000)^0.25)	Empirical	[7,17]
8	Bone marrow	Volume (t = 6 mm)	0.015 + 2×10^{-8}f	50 + 2500 / (1 + $(f / 20000)^2$) + 3000 · δ(f = 0)	Empirical	[7,8]
9	Epineurium	Layered Material Stack (t = 0.1 mm for cutaneous nerve; t = 0.374 mm for muscle nerve)	0.018 (constant)	9.4×10^{-6} (constant)	Resistive	[12]
10	Perineurium	Layered Material Stack (t = 0.014 mm)	0.05 + (0.016 − 0.05) / (1 + (2πf · 8×10^{-6})^0.8)	2000 + 2.5×10^5 / (1 + (2πf · 8×10^{-6})^0.8)	Cole-Cole	[12]

No.	Material	Role	Conductivity σ(f) [S/m]	Relative Permittivity $\varepsilon_r(f)$	Model Type	Ref.
11	Endoneurium (⊥)	Volume (d = 1.772 mm for cutaneous nerve; d = 3.225 mm for muscle nerve)	0.2 (constant)	$4000 + 2.5\times10^{5}$ / (1 + ($2\pi f \cdot 8\times10^{-6}$)^0.8)	Cole-Cole	[12]
12	Endoneurium (∥)	Volume (d = 1.772 mm for cutaneous nerve; d = 3.225 mm for muscle nerve)	1.03 (constant)	$4000 + 2.5\times10^{5}$ / (1 + ($2\pi f \cdot 8\times10^{-6}$)^0.8)	Cole-Cole	[12]
13	Blood	Volume (d = 4 mm)	Three-dispersion Cole-Cole (Eq. 18 in the main text)	Three-dispersion Cole-Cole (Eq. 19 in the main text)	Cole-Cole (α, β, γ)	[18]
14	Air	Volume (t = 4 mm)	5×10^{-13} (constant)	1 (constant)	Constant	COMSOL Multiphysics
15	Silver electrode	Volume	61.6×10^{6} (constant)	1 (constant)	Constant	COMSOL Multiphysics

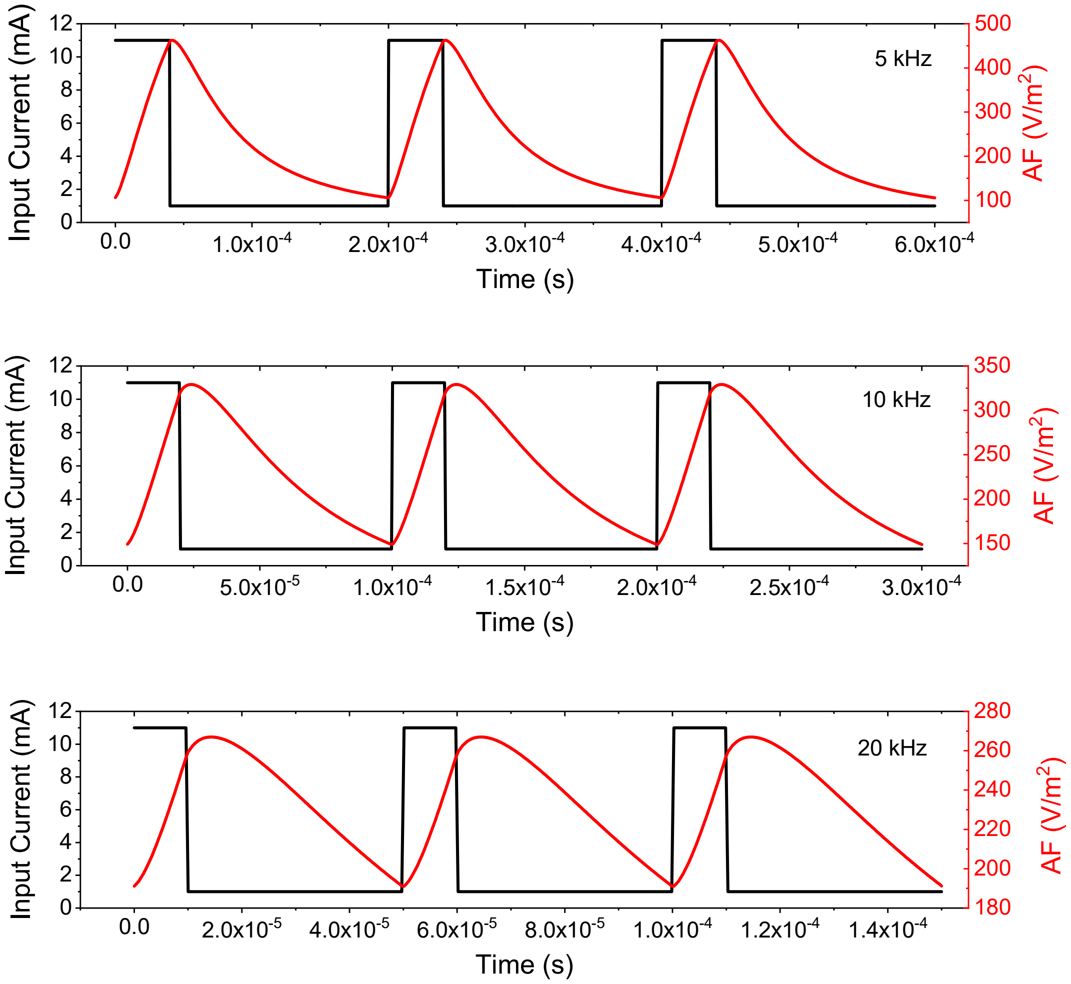

Figure S1. Temporal evolution of neural activation function in response to monophasic square-wave stimulation at different frequencies. Each panel displays the input current waveform (black) and the corresponding time-domain response of the activation function, $\partial^2\Phi/\partial z^2$ (red), computed for a representative cutaneous nerve (Radial Nerve). Stimulation parameters: baseline current = 1 mA, average current = 3 mA, duty cycle = 20%. (Top) Carrier frequency = 5 kHz; (Middle) 10 kHz; (Bottom) 20 kHz.

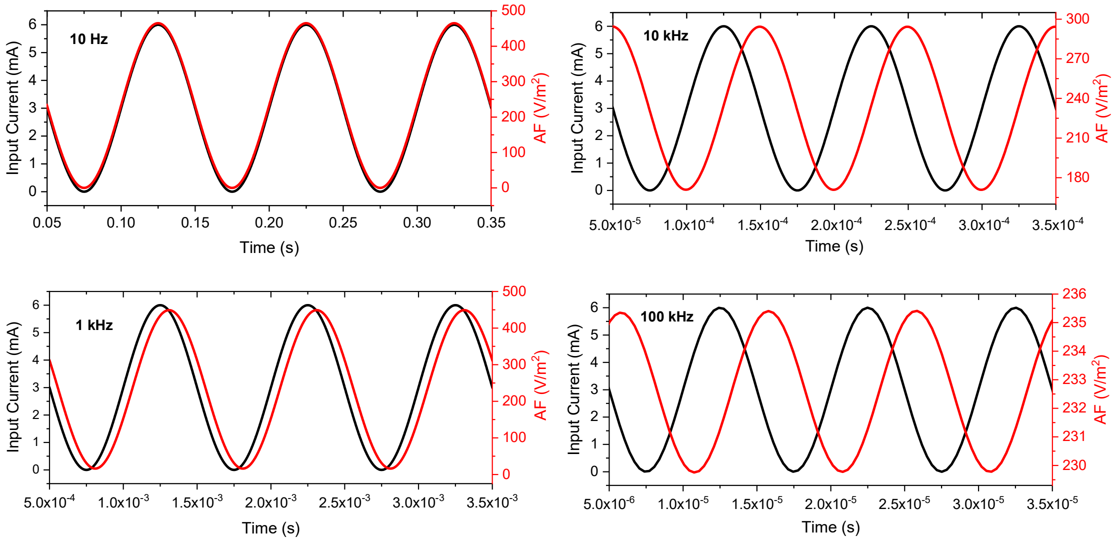

Figure S2. Frequency-dependent attenuation and phase shift in neural response to monophasic sine-wave stimulation. The stimulus is a 3 mA sine wave superimposed on a 3 mA DC offset (net 3 mA). As frequency increases from 10 Hz to 100 kHz, the response amplitude decreases progressively and the phase delay between the stimulus and response increases. Results shown for the Radial nerve.

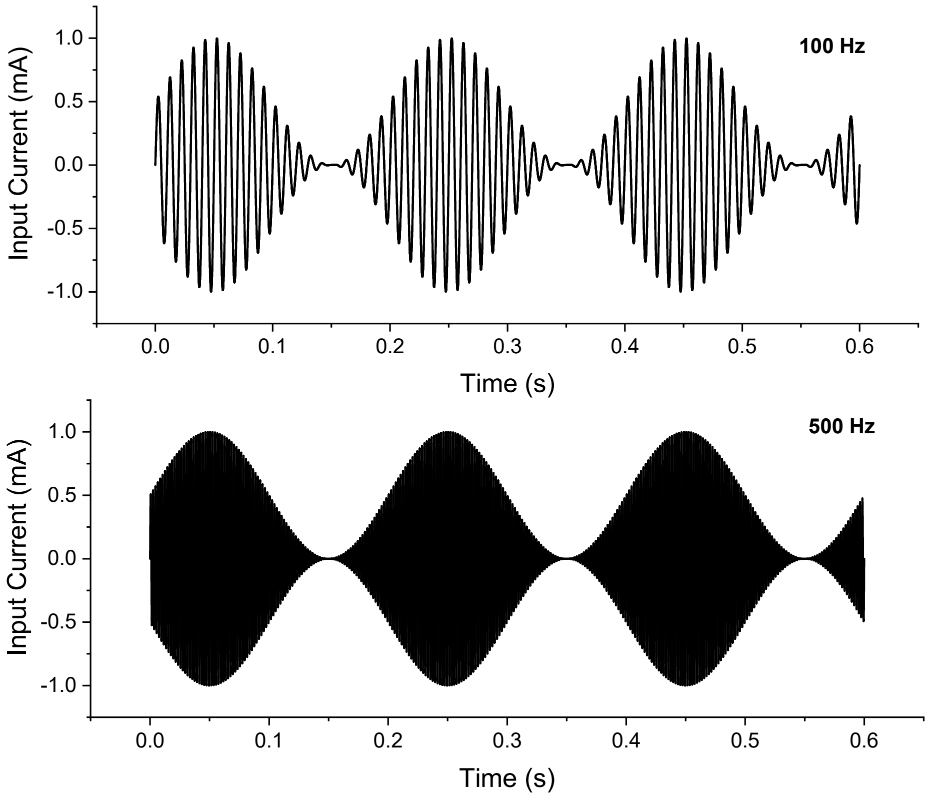

Figure S3. Waveform illustration of amplitude-modulated (AM) sine-wave stimulation. The figure displays the time-domain shape of the input current for two representative AM waveforms. In both panels, the modulation frequency is fixed at 5 Hz and the signal amplitude is 1 mA. (Top) Carrier frequency = 100 Hz. (Bottom) Carrier frequency = 500 Hz. The envelope of the waveform reflects the 5 Hz modulation, while the rapid oscillations correspond to the respective carrier frequency.

Higher carrier frequencies (e.g., 1 kHz, 2 kHz) produce waveforms with oscillations too dense to be clearly resolved in a time-domain plot at this scale; their overall envelope, however, follows the same modulation pattern. These waveforms were used to simulate the neural response and analyze the effect of carrier frequency on stimulation intensity (see Figure 5a).

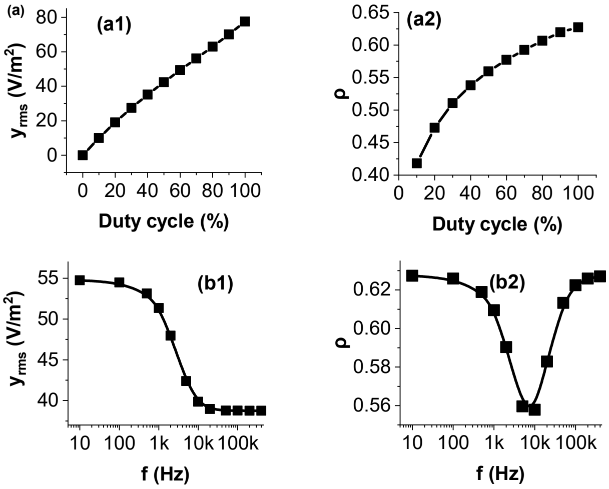

Figure S4. Supplementary parametric study of pulse width and frequency on the Radial nerve with fixed stimulus amplitude (1 mA). (a1) RMS stimulation intensity as a function of duty cycle (f = 5 kHz); (a2) corresponding penetration ratio ρ = yRadial / ySecond_cutaneous. (b1) RMS stimulation intensity as a function of frequency (duty cycle = 50 %); (b2) corresponding penetration ratio ρ. Total current was not held constant in these supplementary experiments; only the peak amplitude was fixed.

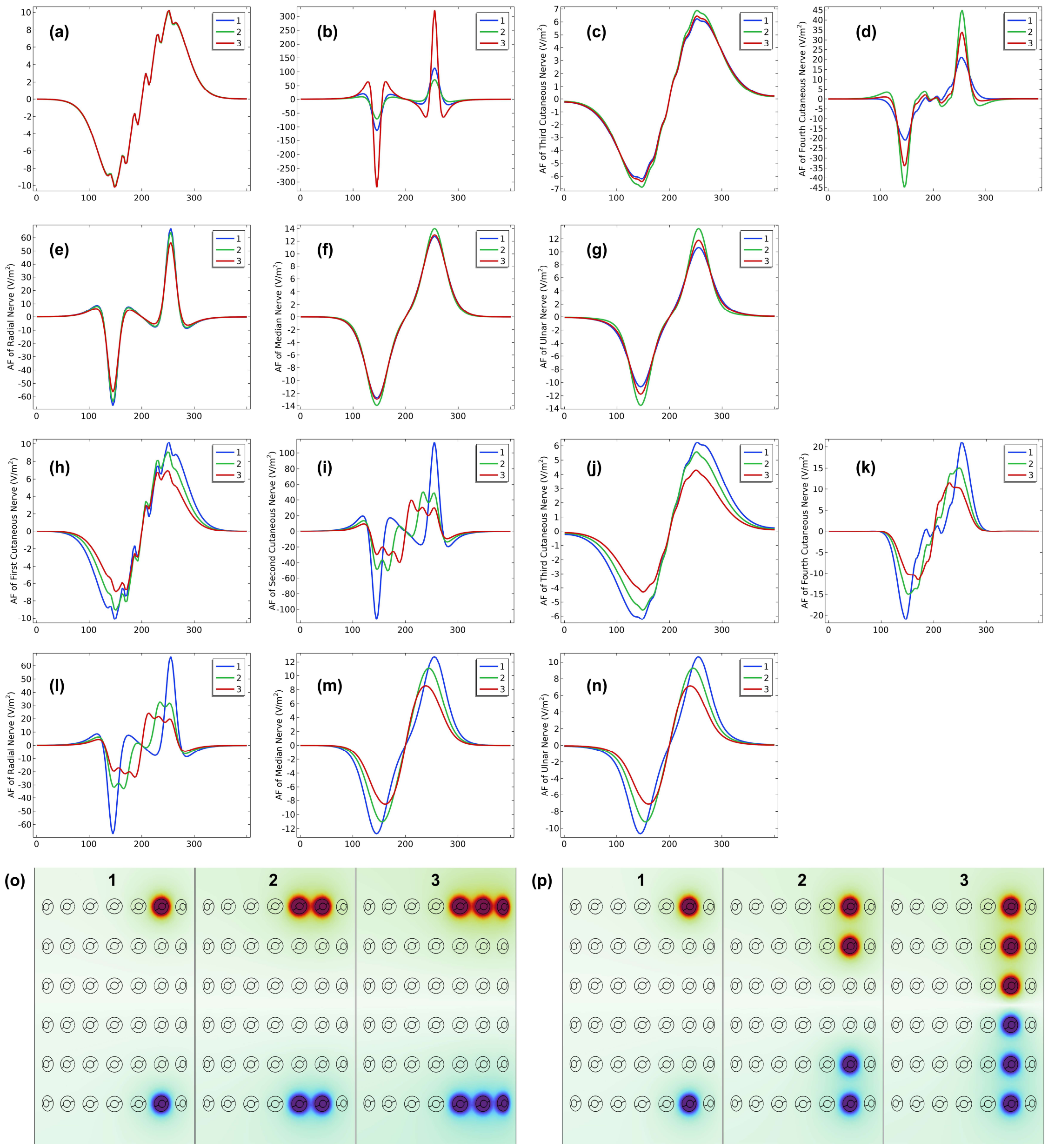

Figure S5. (a–n) Longitudinal distribution of the activating function along the forearm (0–400 mm) for each of the seven nerves under transverse (a–g) and longitudinal (h–n) array configurations, with the number of active electrode pairs varying from 1 to 3 (total current held constant). (a–g) Transverse array expansion: (a) First cutaneous nerve, (b) Second cutaneous nerve, (c) Third cutaneous nerve, (d) Fourth cutaneous nerve, (e) Radial nerve, (f) Median nerve, (g) Ulnar nerve. (h–n) Longitudinal array expansion: (h) First cutaneous nerve, (i) Second cutaneous nerve, (j) Third cutaneous nerve, (k) Fourth cutaneous nerve, (l) Radial nerve, (m) Median nerve, (n) Ulnar nerve.

(o) Schematic diagrams of transverse array configurations with 1, 2, and 3 active electrode pairs (labeled 1, 2, and 3, respectively). (p) Schematic diagrams of longitudinal array configurations with 1, 2, and 3 active electrode pairs (labeled 1, 2, and 3, respectively).
